# Rational Design of Chemically Controlled Antibodies and Protein Therapeutics

**DOI:** 10.1021/acschembio.3c00012

**Published:** 2023-05-30

**Authors:** Anthony Marchand, Lucia Bonati, Sailan Shui, Leo Scheller, Pablo Gainza, Stéphane Rosset, Sandrine Georgeon, Li Tang, Bruno E. Correia

**Affiliations:** † Laboratory of Protein Design and Immunoengineering, Institute of Bioengineering, Ecole Polytechnique Fédérale de Lausanne (EPFL), CH-1015 Lausanne, Switzerland; ‡ Laboratory of Biomaterials for Immunoengineering, Institute of Bioengineering, Ecole Polytechnique Fédérale de Lausanne (EPFL), CH-1015 Lausanne, Switzerland

## Abstract

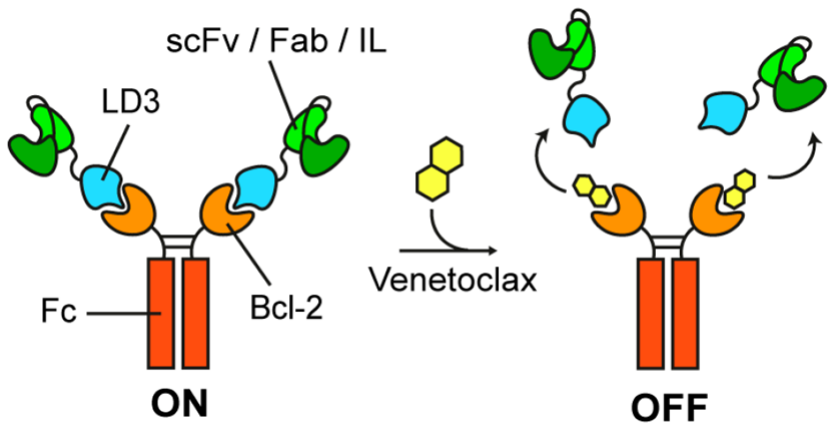

Protein-based therapeutics, such as monoclonal antibodies and cytokines, are important therapies for various pathophysiological conditions such as oncology, autoimmune disorders, and viral infections. However, the wide application of such protein therapeutics is often hindered by dose-limiting toxicities and adverse effects, namely, cytokine storm syndrome, organ failure, and others. Therefore, spatiotemporal control of the activities of these proteins is crucial to further expand their application. Here, we report the design and application of small-molecule-controlled switchable protein therapeutics by taking advantage of a previously engineered OFF-switch system. We used the Rosetta modeling suite to computationally optimize the affinity between B-cell lymphoma 2 (Bcl-2) protein and a previously developed computationally designed protein partner (LD3) to obtain a fast and efficient heterodimer disruption upon the addition of a competing drug (Venetoclax). The incorporation of the engineered OFF-switch system into anti-CTLA4, anti-HER2 antibodies, or an Fc-fused IL-15 cytokine demonstrated an efficient disruption in vitro, as well as fast clearance in vivo upon the addition of the competing drug Venetoclax. These results provide a proof-of-concept for the rational design of controllable biologics by introducing a drug-induced OFF-switch into existing protein-based therapeutics.

Protein-based therapeutics, such as monoclonal antibodies (mAbs) and cytokines, have been shown to mediate potent antitumor effects and are the fastest growing group of therapeutics.^1,2^ Nevertheless, their therapeutic use is limited by systemic toxicities arising from excessive immune and inflammatory responses and by off-target effects.^3,4^ Innovative engineering strategies have been applied to increase safety through localized activity of the therapeutic^5−7^ or drug-induced ON-switch system.^8^ However, none of these approaches allows the direct OFF-switch control of the therapeutics’ activity with an external trigger that can be applied as desired. A system that allows the spatiotemporal control of biological activities upon administration of clinically approved small molecules represents a promising strategy to increase protein therapeutics’ safety profile. Several prior studies focused on modulating protein–protein interactions (PPIs) using small molecules to trigger either disruption or dimerization.^9−13^ We previously reported a novel chemically disruptable heterodimer composed (CDH) of a BH3-motif grafted and computationally improved protein (LD3) binding to B-cell lymphoma-extra large (Bcl-XL) or B-cell lymphoma 2 protein (Bcl-2) with high affinity.^14,15^ The heterodimers can be disrupted by A-1155463 and Venetoclax, respectively. However, this approach has never been used to control the activity of a soluble protein therapeutic. Here, we computationally optimized the interface of the CDH for enhanced drug sensitivity and faster disruption. We used the optimized CDH to disrupt the Fc region from a therapeutic protein to control its half-life. Our results demonstrate the potential of designed OFF-switches for generating biologics with enhanced safety and broader applications.

To generate switchable antibodies (SwAbs), we placed the LD3:Bcl-2 complex, which can be disrupted by Venetoclax, between the epitope-binding region and the fragment crystallizable (Fc) region of the antibody (Figure 1A). Fc regions are crucial for antibodies as they provide important features such as (i) a longer half-life *in vivo*,^16^ (ii) an increased avidity effect due to the dimerization,^17^ and (iii) an ability to trigger effector functions.^18^ We hypothesized that the addition of Venetoclax would compete for the LD3-binding site on Bcl-2 and trigger disruption between the two components. As a result, the epitope-binding domain would lose the advantages provided by the Fc region, leading to an indirect OFF-switch of the biological activity.

**Figure 1:**
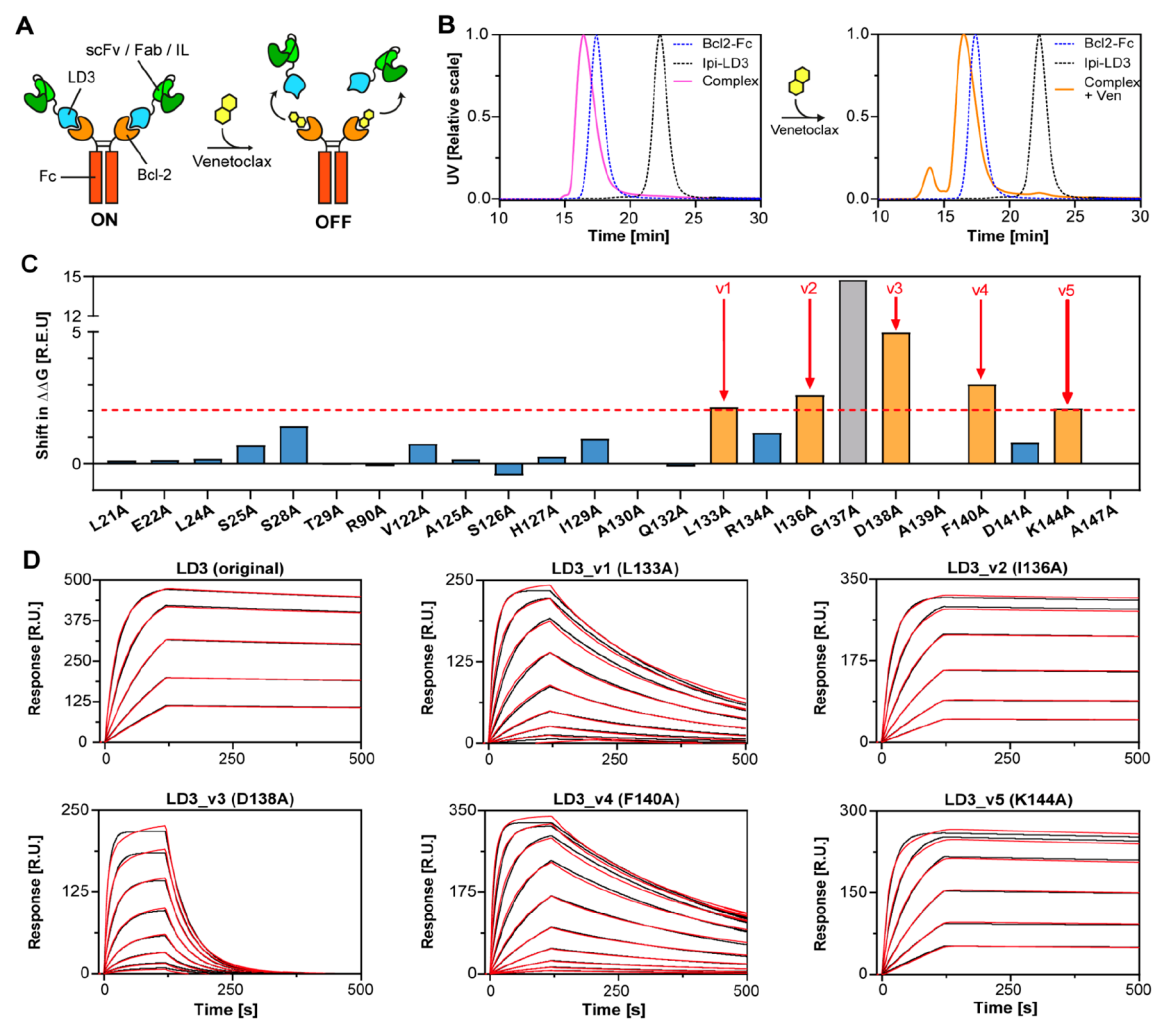
Computational design and improvement of a switchable antibody system. (A) Schematic representation of the switchable antibody system. A single-chain variable fragment (scFv) or fragment antigen-binding region (Fab) or interleukin (IL) is fused to a computational design (LD3) with high affinity to the Fc-fused Bcl-2. The addition of Venetoclax binds to the LD3-binding site on Bcl-2 and triggers disruption of the switchable antibody. (B) SEC-MALS of an αCTLA4 Fab fused to LD3 (Ipi-LD3, gray dashed line), an Fc-fused Bcl-2 (blue dashed line), the switchable antibody complex (pink, left part), and the switchable antibody complex incubated with Venetoclax (orange, right part). (C) Computational alanine scan obtained with Rosetta. Mutations to alanine giving an increase of the computed binding energy (ΔΔ*G*) of at least two Rosetta energy units (R.E.U.) were considered as variant candidates (orange bars). The G137A mutation was not considered (gray bar). (D) Surface plasmon resonance (SPR) with Bcl-2 binding to different immobilized LD3-variants (v1 to v5). Measurements are indicated in red and fit curves in black. The highest concentration of Bcl-2 starts at 2000 nM for LD3 variants 1, 3, and 4 and starts at 500 nM for original LD3 and variants 2 and 5. A 2-fold dilution factor was then applied between each concentration.

We first generated a switchable version of a published αCTLA4 fragment antigen-binding region (Fab, Ipilimumab)^19^ and tested the disruption efficiency by detecting the complex and monomeric components by size-exclusion chromatography combined with multiangle light scattering (SEC-MALS). However, Venetoclax did not trigger detectable SwAb disruption as monomeric components were not observed (Figure 1B, Table S1). Similar observations were obtained when replacing the therapeutic moiety fused to LD3 by an αHER2 single-chain variable fragment (scFv) or a mouse interleukin-15 superagonist (IL-15SA; Figure S1). We therefore hypothesized that the low-nanomolar affinity of the LD3:Bcl-2 complex (Table 1) does not allow an efficient competition by the drug, most probably due to the slow dissociation rate (*k*_off_) that restricts the opportunity of the drug to displace the LD3 binder. With these considerations, we aimed to further engineer LD3 for reduced affinity for Bcl-2. We used the protein modeling framework Rosetta to conduct a computational alanine scan on all LD3 interface residues to highlight alanine mutants with increased computed binding energy (ΔΔ*G*; Figure 1C). All mutations to alanine increasing the ΔΔ*G* by 2 Rosetta energy units (R.E.U.) were considered as potential LD3 variant candidates (v1 to v5), except for G137A, which introduces a steric clash likely to be considerably deleterious for binding.

**Table 1:** Summary Table of the Affinities Surface Plasmon Resonance Data of the Different LD3 Variants^a^

	LD3 (original)	LD3_v1	LD3_v2	LD3_v3	LD3_v4	LD3_v5
*k*_on_ [10^4^ M^–1^ s^–1^]	29.1 ± 2.7	4.08 ± 0.34	36.0 ± 3.15	15.4 ± 0.26	24.0 ± 2.12	45.5 ± 3.70
*k*_off_ [10^–4^ s^–1^]	1.23 ± 0.65	26.3 ± 7.75	0.89 ± 0.44	184 ± 30.4	19.7 ± 5.45	0.66 ± 0.67
*K*_D_ [nM]	1.40 ± 0.86	65.8 ± 25.1	0.74 ± 0.28	358 ± 55.8	27.3 ± 15.9	0.46 ± 0.41

a Data were collected using SPR showing the association rate (*k*_on_), dissociation rate (*k*_off_), and dissociation constant (*K*_D_) of the original LD3 and the different variants obtained by computational alanine scanning. Data represent mean ± standard deviation from three independent experiments.

The remaining five LD3 variants were expressed, purified, and tested by surface plasmon resonance (SPR) for binding Bcl-2 compared to the original LD3 protein (Figure 1C,D, Table 1, and Figure S2). We sought to find variants with a slightly decreased dissociation rate (*k*_off_) compared to the original LD3, but with an unperturbed association rate (*k*_on_). Variants 2 (I136A) and 5 (K144A) showed only minor differences from the original LD3 and were not further considered. Variant 3 (D138A) had the highest destabilization effect, which is consistent with the high ΔΔ*G* difference predicted by the alanine scan. Both variants 1 (L133A) and 4 (F140A) showed similar mild decreases in dissociation rates; however, variant 4 had a less affected association rate and was therefore chosen as a lead candidate for the switchable antibody system. We used LD3 variant 4 (LD3_v4) to generate an improved version of the switchable Ipilimumab-based αCTLA4 antibody by fusing the Ipilimumab Fab to LD3_v4. After complex formation with Bcl2-Fc, we assessed the switchability using SEC-MALS as described above. While only 3% (*m*_uncomplex_/*m*_total_) of the switchable antibodies were disrupted on SEC-MALS upon Venetoclax treatment with the original LD3 protein, more than 90% of the complex was efficiently disrupted with LD3_v4 (Figure 2A, Table S1). Similarly, we noticed comparable results with the αHER2 and IL-15SA switchable therapeutics, demonstrating the modularity of the system (Figure S3). We evaluated disruption kinetics by biolayer interferometry (BLI) and detected 30% disruption at the highest tested concentration of Venetoclax (10 μM) after 200 s (Figure 2B). During that time, the switchable antibody complex remained stable in solution without the addition of Venetoclax.

**Figure 2:**
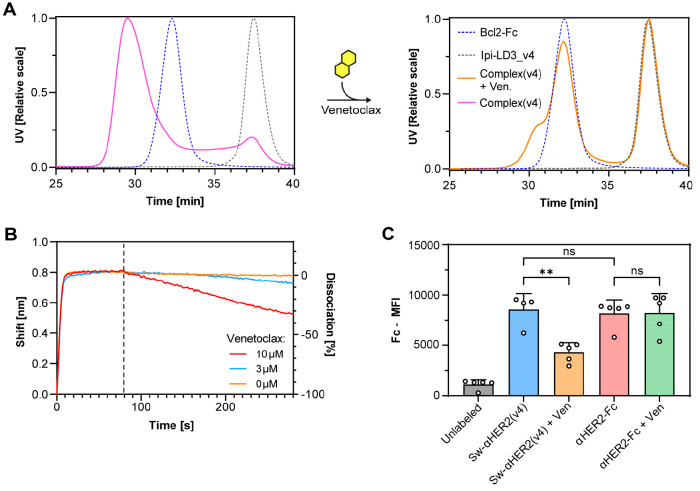
Disruption efficiency of a switchable antibody with LD3_v4. (A) SEC-MALS of a Bcl2-Fc alone (blue dashed line), an αCTLA4 Fab (Ipilimumab) fused to LD3 variant 4 (gray dashed line) and the switchable antibody complex in the absence (pink) and presence (orange) of 100 μM Venetoclax. (B) Biolayer interferometry (BLI) measurements of the switchable anti-CTLA4 antibody with increasing concentration of Venetoclax. (C) Quantification of the mean fluorescence intensity (MFI) measured on the surface of MC38 cells unlabeled or labeled with a switchable or conventional αHer2 antibody (Sw-αHER2 and αHER2-Fc respectively) treated without or with 10 μM Venetoclax. Tukey’s multiple comparisons test, *p* < 0.01 (**), nonsignificant (ns). Data points represent technical replicates with mean and standard deviation.

To confirm these results in a cell-based assay, we substituted the antigen-targeting domain of the SwAb with an αHER2 scFv that allowed the labeling of HER2-expressing cells. We stained MC38-HER2 cells, a murine colon adenocarcinoma cell line stably expressing HER2, with the switchable αHER2 antibody and treated the cells with or without Venetoclax (Figure 2C and Figure S4). One hour after adding Venetoclax, the Fc fragment detected on the MC38 cell surface decreased by 2-fold. Among other possibilities, the reduced Venetoclax-induced antibody disruption might be explained by the avidity provided by the two Fabs binding simultaneously, which may reduce drug sensitivity. The switchable αHER2 antibody showed similar binding to MC38-HER2 cells compared to a conventional αHER2 antibody, which did not respond to Venetoclax, and no disruption was observed when using the original LD3 protein (Figure S4). Altogether, these results confirm the improved switchability of the engineered antibody.

We next tested the function of the engineered switchable proteins *in vitro* and *in vivo* by measuring cell proliferation and the half-life in mice blood. To do so, we extended the strategy to the generation of switchable cytokines. We chose mouse IL-15 superagonist (IL-15SA) and generated switchable IL-15SA (SwIL-15SA) by fusing IL-15 and the IL-15 receptor α domain (IL-15Rα) to LD3 assembled with Bcl2-Fc (Figure 3A). To assess the functionality of SwIL-15SA, we stimulated primary murine T cells *ex vivo* with either IL-15SA or SwIL-15SA and measured cell proliferation. Proliferation of murine primary T cells induced by SwIL-15SA was comparable to conventional IL-15SA, indicating that fusing LD3 to the sushi domain of IL-15Rα did not hinder its functionality (Figure 3B).

**Figure 3:**
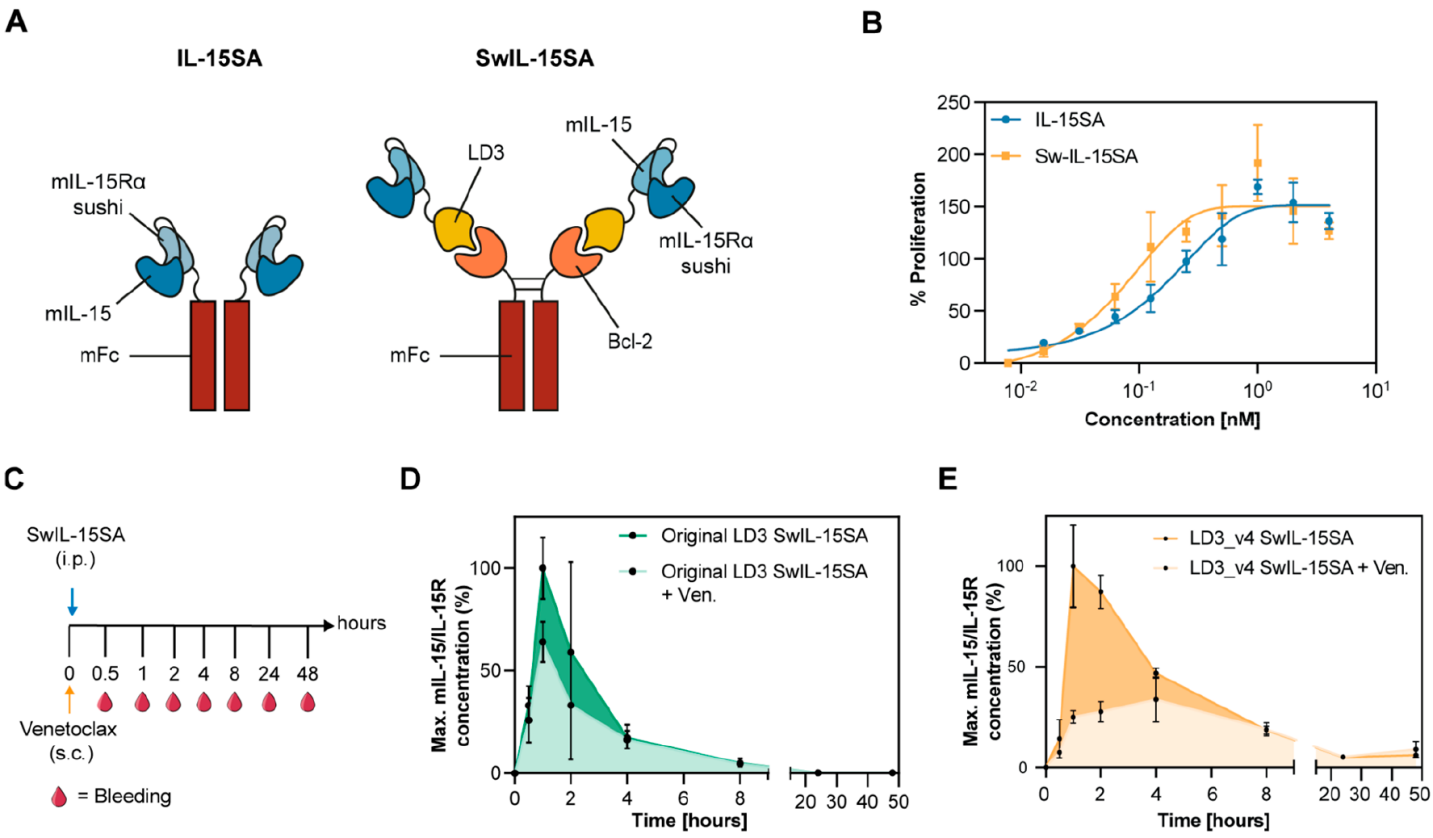
Functional assessment and *in vivo* studies using an Fc-fused switchable cytokine. (A) Schematic representation of the switchable interleukin system. In IL-15SA, the sushi domain of mouse IL-15Rα is fused to mIL-15 binding a mouse Fc (left). In SwIL-15SA, the sushi domain of mouse IL-15Rα is fused to the optimized LD3 binding to mouse Fc-fused Bcl-2 (right). (B) Activated mouse T cell proliferation in response to IL-15SA or SwIL-15SA. (C) C57BL/6 mice were first injected subcutaneously (s.c.) with Venetoclax (25.0 mg/kg) and subsequently injected intraperitoneally (i.p.) with 100 pmol of SwIL-15SA. Mice were bled over time 0.5, 1, 2, 4, 8, 24, and 48 h after treatment. (D) Pharmacokinetic properties of SwIL-15SA composed of the IL-15/IL-15R complex fused to the original LD3 with (light green) or without (dark green) the administration of Venetoclax. E. Pharmacokinetic properties of SwIL-15SA composed of the IL-15/IL-15R complex fused to LD3_v4 with (light orange) or without (dark orange) the administration of Venetoclax.

In a second step, we assessed the switchability of SwIL-15SA *in vivo*. C57BL/6 mice were first injected subcutaneously (s.c.) with or without Venetoclax and then intraperitoneally (i.p.) with SwIL-15SA containing the original LD3. Mice were bled overtime after treatment, and IL-15/IL-15R complex concentration was measured by enzyme-linked immunosorbent assay (ELISA; Figure 3C). The IL-15/IL-15R complex concentration in the blood of mice treated with Venetoclax peaked at 64% of the maximum IL-15/IL-15R concentration of the control group, confirming that Venetoclax administration does not lead to the efficient disruption of the original LD3:Bcl-2 complex, as demonstrated in *in vitro* experiments (Figure 3D and Figure S5). To investigate whether the affinity of the Bcl-2:LD3 complex could provide a parameter to tune the switchability efficiency of the system, we further tested a variant of SwIL-15SA being composed of the IL-15 sushi domain fused to LD3_v4. Here, blood concentrations in control mice peaked at 1 h after injection and then decreased over time (Figure 3E and Figure S5). Unlike the control group, in mice treated with Venetoclax, the IL-15/IL-15R complex concentration reached only about 25% of the maximum IL-15/IL-15R concentration of the control group. This observation suggests that the disruption efficiency and the half-life of the system can be tuned with the affinity of the Bcl-2:LD3 complex. Overall, these results show that Venetoclax disrupts the interaction between Bcl-2 and LD3, leading to the fast clearance of monomeric IL-15/IL-15R-LD3 *in vivo*.

Altogether, we show a modular and generalizable OFF-switch approach for the design of safe antibody and cytokine therapeutics by introducing a chemically disruptable heterodimer between the therapeutic domain and the Fc moiety. Loss of the Fc fragment leads to a decrease of the avidity effect and a drastic reduction of the protein half-life. We took advantage of a previously designed CDH that can be competed with a clinically approved drug, Venetoclax, which makes it a good candidate for translational applications. Of note, one strength of our system is its modularity with the ease of adapting it to several therapeutic proteins by exchanging the therapeutic domain fused to LD3. But the large size of the protein complex (of about ∼250 kDa for a switchable antibody, compared to ∼150 kDa for a normal antibody) may limit tissue penetration.^20^ However, for highly toxic therapies, such as immunostimulatory therapies, these limitations would be outweighed by the improved safety profile. Our presented workflow to reduce heterodimer affinity to increase drug sensitivity can likely be readily extended to other examples of CDHs. These types of switchable biologics could serve as a basis for safer biologics for therapeutic use.

## Methods

### Computational Design

The previously solved crystal structure of Bcl-2 in complex with LD3 was used for computational modeling (PDB ID: 6IWB). Using the Rosetta modeling suite, the pose was relaxed with the “FastRelax” mover, before the computational alanine scan was performed using an “Alascan” filter. Residues where a mutation to alanine led to an increase of the computed binding energy of >2 Rosetta energy units (R.E.U.) were considered as potential candidates to lower the affinity of LD3 for Bcl2. Mutations exceeding 5 R.E.U. were not considered due to the introduction of clashes that may abrogate binding.

### Protein Expression and Purification

The engineered IL-15SA construct (gWIZ-mIL-15SA) was a gift from D. J. Irvine (MIT). IL-15SA contains a mouse IL-15 fused at the C terminus of the sushi domain of a mouse IL-15Rα, which is next fused at the C terminus with a mouse IgG2c Fc. A previously optimized version of Bcl-2^21^ was fused to either a human IgG or mouse IgG2 Fc-fragment (see Table S2). Switchable antibodies were composed of either a previously published αCTLA4 antibody^22^ Ipilimumab as a Fab or an αHER2 4D5 clone^23^ as an scFv fused to LD3 protein N-terminal with a (GGGS)3-linker. As a switchable cytokine, we used a fusion protein composed of mouse IL-15 C-terminally fused to the IL-15 receptor α domain (IL-15Rα), itself fused to the LD3 variant C-terminal with a (GGGS)3-linker. DNA sequences were ordered from Twist Bioscience and Gibson cloning used to clone into bacterial (pET11) or mammalian (pHLSec) expression vectors. Mammalian expressions were performed using the Expi293TM expression system from Thermo Fisher Scientific. The supernatant was collected 6 days post transfection, filtered, and purified. *E. coli* expressions were performed using BL21 (DE3) cells and IPTG induction (1 mM at OD 0.6–0.8) and growth overnight at 16–18 °C. Pellets were lysed in lysis buffer (50 mM Tris, pH 7.5, 500 mM NaCl, 5% glycerol, 1 mg mL^–1^ lysozyme, 1 mM PMSF, and 1 μg mL^–1^ DNase) with sonication, and the lysate clarified and and purified. Proteins were then purified using an ÄKTA pure system (GE healthcare) with Ni-NTA affinity columns followed by size exclusion chromatography with PBS.

### Surface Plasmon Resonance

SPR measurements were performed on a Biacore 8K (GE Healthcare) with HBS-EP+ as a running buffer (10 mM HEPES at pH 7.4, 150 mM NaCl, 3 mM EDTA, 0.005% v/v Surfactant P20, GE Healthcare). Original LD3 and mutants were immobilized on a CM5 chip (GE Healthcare #29104988) via amine coupling. 500–1000 response units (RU) were immobilized, and Bcl-2 was injected as an analyte in serial dilutions. The flow rate was 30 μL/min for a contact time of 120 s followed by 400 s of dissociation time. After each injection, the surface was regenerated using 50 mM NaOH. SPR data were fit with a 1:1 Langmuir binding model within the Biacore 8K analysis software (GE Healthcare #29310604).

### Biolayer Interferometry (BLI)

Measurements were performed on a Gator BLI system. The running buffer was PBS. Fc-tagged Bcl-2 was diluted to 5 μg/mL and immobilized on antihuman IgG tips for 80 s (1–2 nm immobilized). The loaded tips were then dipped into 500 nM LD3-fused Ipilimumab Fab (or PBS for the reference) for 80 s and then in different concentrations of Venetoclax (10, 3, and 0 μM) diluted in PBS for 210 s. Each measurement was subtracted with the reference (channel with Fc-fused Bcl2 immobilized, no associated LD3, and a corresponding concentration of Venetoclax diluted in PBS).

### Size Exclusion Chromatography Multiangle Light Scattering (SEC-MALS)

Size exclusion chromatography with an online multiangle light scattering device (miniDAWN TREOS, Wyatt) was used to determine the oligomeric state and molecular weight for the switchable antibodies in solution. Purified LD3-Fab and Bcl2-Fc proteins were mixed with a 2:1 molar ratio and incubated at RT for 5 min to form a complex. Assembled complexes received 100 μM Venetoclax or PBS and were incubated 1 h at 37 °C. The final concentration was approximately 1 mg mL^–1^ in PBS (pH 7.4), and 100 μL of the sample was injected into a Superdex 200 300/10 GL column (GE Healthcare) with a flow rate of 0.5 mL/min. UV280 and light scattering signals were recorded. Molecular weight was determined using the ASTRA software (version 6.1, Wyatt).

### *In Vitro* Cell Binding Assay

100 000 HER2-transduced MC38 mouse colon cancer cells were collected in a tube. Purified HER2-specific LD3-Fab and Bcl-2-Fc proteins were mixed at a 2:1 ratio and incubated at RT for 5 min to form a complex. MC38-HER2+ cells were then stained with αHER2 SwAb at concentrations of 100 nM and incubated at 4 °C for 30 min. An Fc-fused αHER2 (αHER2-Fc) was used as a positive control. Cells were washed twice with FACS buffer (PBS containing bovine serum albumin, 0.2% (w/v)), and 10 μM Venetoclax was added to the cells and incubated at 37 °C for 1 h. Afterwards, cells were washed and stained with an A647-conjugated antihuman Fc antibody (BioLegend, ref. 409319) at 4 °C for 30 min. Cells were then washed, stained with 4′,6-diamidino-2-phenylindole (DAPI; Sigma-Aldrich), and analyzed via FACS.

### T-Cell Proliferation Assay

Activated Pmel T cells were collected by centrifugation, resuspended in mouse T-cell media, and seeded at a density of 10 000 T cells/well in a 96-well flat bottom tissue culture plate. T cell growth was stimulated by the addition of serial dilutions of IL-15SA or SwIL-15SA to a total volume of 100 μL and cultured for 48 h at 37 °C. On day 2, cells were collected, washed once with FACS buffer, and stained with DAPI. Cell counts for each condition were quantified by FACS using the Attune NxT flow cytometer (Invitrogen/Thermo Fisher Scientific).

### Animal Studies

6–8 week-old female C57BL/6 mice were purchased from Charles River Laboratories and maintained in the animal core facility [Center of Phenogenomics (CPG)] of École Polytechnique Fédérale de Lausanne (EPFL). All experiments were conducted according to the Swiss Federal Veterinary Office guidelines and were approved by the Cantonal Veterinary Office. In evaluating the switchability potential of SwIL-15SA, C57BL/6 mice were injected subcutaneously with 100 μL of Venetoclax dissolved at 25 mg/kg in a solution of saline and 2% dimethyl sulfoxide (DMSO). Afterward, the animals were injected intraperitoneally with 100 pmol of SwIL-15SA in 100 μL and bled over time at 0.5, 1, 2, 4, 8, 24, and 48 h after treatment. The IL-15/IL-15R complex concentration in blood was quantified using a commercial enzyme-linked immunosorbent assay (ELISA) kit following the manufacturer’s instructions (Thermo Fisher Scientific, 88-7215-88).

## Supporting Information Available

Supplementary figures of *in vitro* and *in vivo* data and protein sequences table (PDF)
