# Supplementary material for: Rational Design of Chemically Controlled Antibodies and Protein Therapeutics
Source: ACS Chem Biol. 2023 May 30;18(6):1259–65. doi: 10.1021/acschembio.3c00012 (PMC10278067; doi:10.1021/acschembio.3c00012)
Supplement: Supplementary file 1 — cb3c00012_si_001.pdf [file cb3c00012_si_001.pdf]

Supporting Information

Rational design of chemically controlled antibodies and protein therapeutics

Anthony Marchand[1,‡], Lucia Bonati[1,2,‡], Sailan Shui[1], Leo Scheller[1], Pablo Gainza[1], Stéphane Rosset[1], Sandrine Georgeon[1], Li Tang[2]* & Bruno E. Correia[1]*

[1] Laboratory of Protein Design and Immunoengineering, Institute of Bioengineering, Ecole polytechnique fédérale de Lausanne (EPFL), CH-1015 Lausanne, Switzerland

[2] Laboratory of Biomaterials for Immunoengineering, Institute of Bioengineering, Ecole polytechnique fédérale de Lausanne (EPFL), CH-1015 Lausanne, Switzerland

‡ Equal contribution

* Corresponding author: li.tang@epfl.ch & bruno.correia@epfl.ch

---

Table of Contents

A. Supplemental Figures

Figure S1: Size exclusion chromatography of αHER2 antibody and mouse interleukin 15 fused to the original LD3 protein

Figure S2: Kinetic measurements of the different LD3 variants

Figure S3: Size exclusion chromatography of αHER2 antibody and mouse interleukin 15 fused to the LD3_v4 protein

Figure S4: HER2-Overexpressing cells MC38 labeling and controls

Figure S5: *In vivo* studies using an Fc-fused switchable cytokine (Absolute scale)

B. Supplemental Tables

Table S1: Mass fraction of the different SwAb components measured by the SEC-MALS upon Venetoclax treatment.

Table S2: Amino acid sequences of the different proteins used

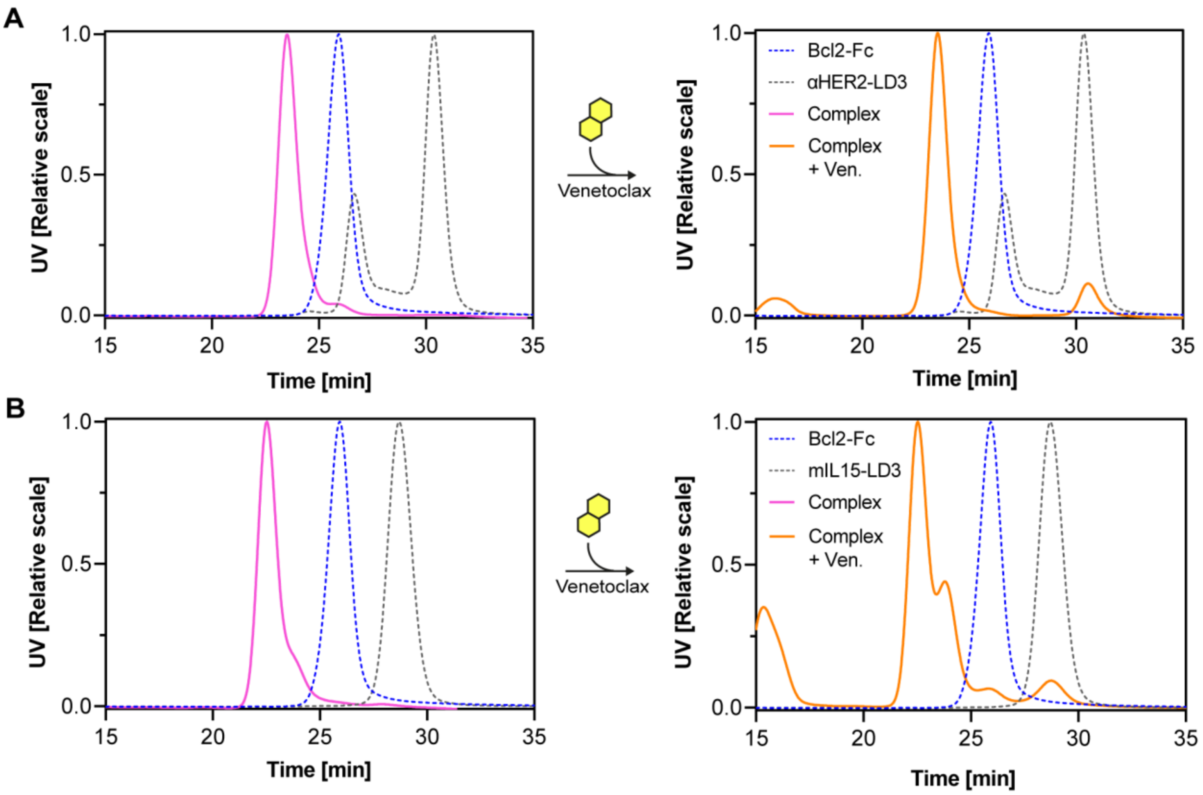

Figure S1: Size exclusion chromatography of αHER2 antibody and mouse interleukin 15 fused to the original LD3 protein. Size exclusion chromatography of an αHER2 single-chain variable fragment (**A**) or a mouse interleukin 15 (**B**) fused to the original LD3. Plots show the switchable protein therapeutic complex in absence (pink) or in presence (orange) of Venetoclax, compared to the Bcl2-Fc (blue, dashed line) or original LD3-fused moiety alone (gray, dashed line)

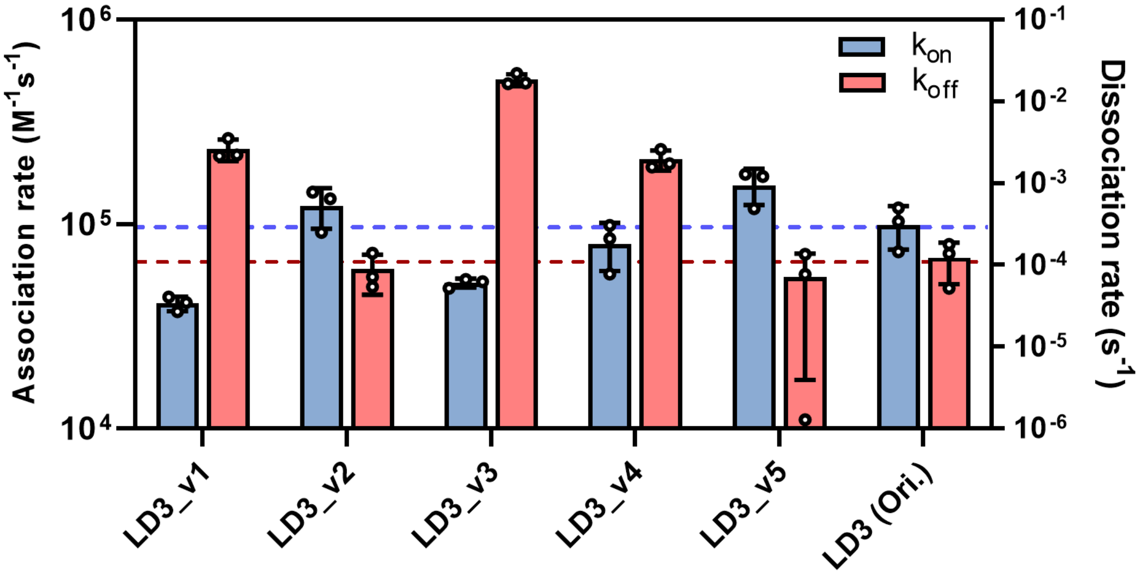

Figure S2: Kinetic measurements of the different LD3 variants. Association rates (k_{on}) are shown with blue bars and dissociation rate (k_{off}) with red bars. Dashed lines show the mean values for the original LD3 (LD3 Ori.) for comparison. Data points represent mean ± standard deviation from three independent experiments.

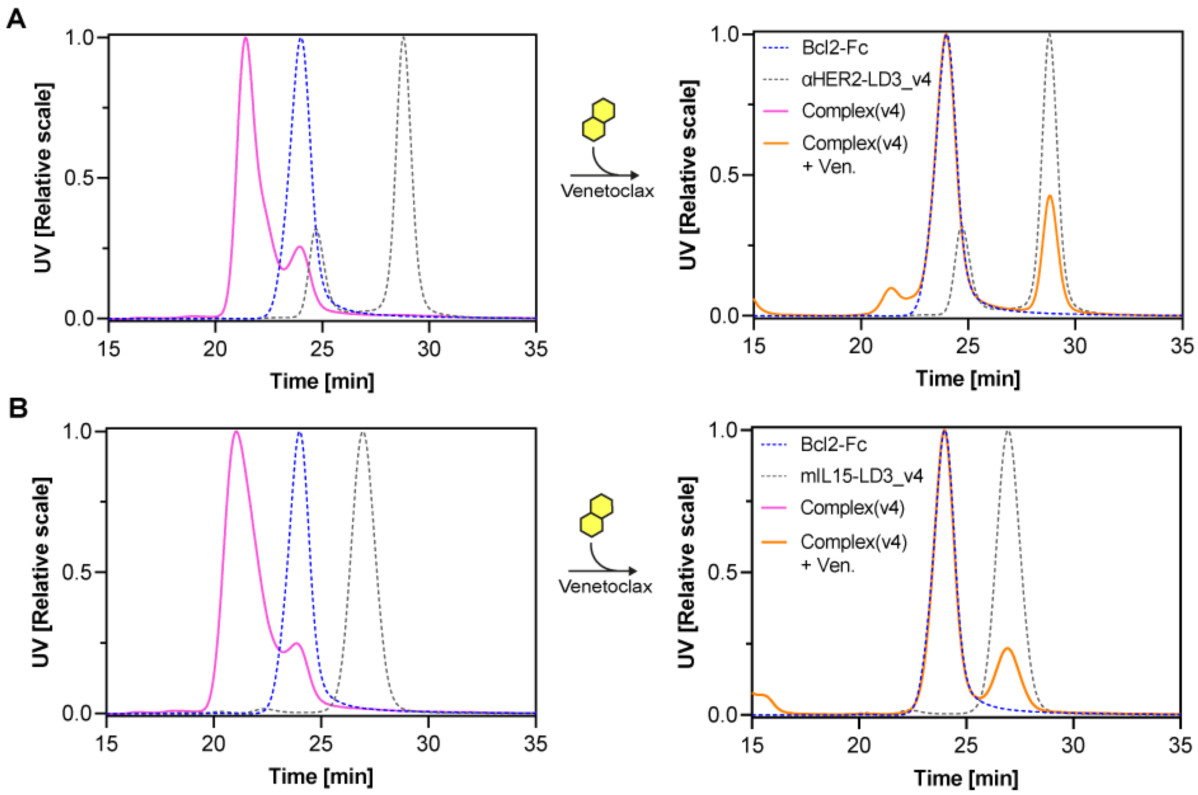

Figure S3: Size exclusion chromatography of αHER2 antibody and mouse interleukin 15 fused to the LD3_v4 protein. Size exclusion chromatography of an αHER2 single-chain variable fragment (**A**) or a mouse interleukin 15 superagonist (IL-15SA) (**B**) fused to LD3_v4. Plots show the switchable protein therapeutic complex in absence (pink) or in presence (orange) of Venetoclax, compared to the Bcl2-Fc (blue, dashed line) or LD3_v4-fused moiety alone (gray, dashed line)

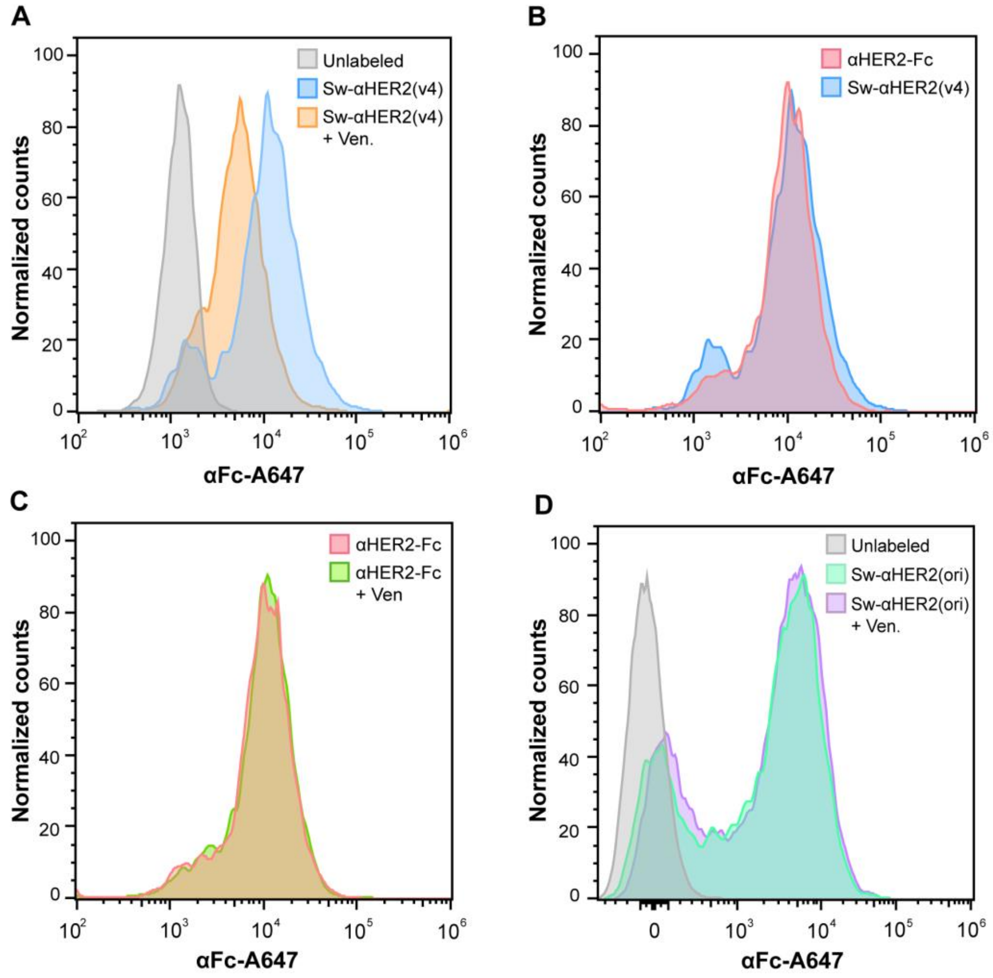

Figure S4: HER2-Overexpressing cells MC38 labeling and controls. A. Histogram of MC38 cells labeled with switchable αHer2 antibody (Sw-αHER2) composed of LD3_v4 in presence or absence of venetoclax (Ven.), **B.** Histogram of MC38 cells labeled with switchable αHer2 antibody (Sw-αHER2) or conventional αHer2 antibody (αHER2-Fc). **C.** Histogram of MC38 cells labeled with conventional αHer2 antibody (αHER2-Fc) in presence or absence of Venetoclax (Ven.) **D.** Histogram of MC38 cells labeled with switchable αHer2 antibody (Sw-αHER2) composed of the original LD3 (ori.) in presence or absence of venetoclax (Ven.)

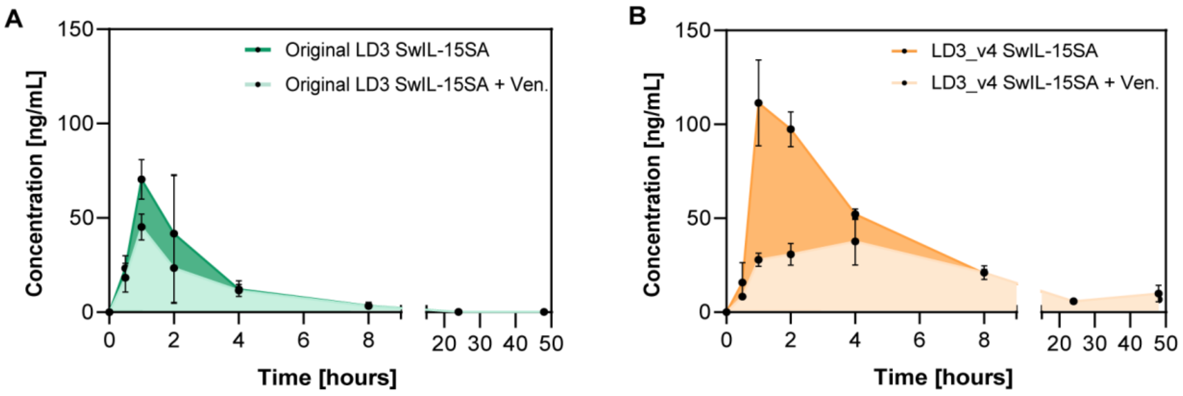

Figure S5: *In vivo* studies using an Fc-fused switchable cytokine (Absolute scale) A. Pharmacokinetic properties of SwIL-15SA composed of the IL-15/IL-15R complex fused to the original LD3 with (light green) or without (dark green) the administration of Venetoclax. **B.** Pharmacokinetic properties of SwIL-15SA composed of the IL-15/IL-15R complex fused to LD3_v4 with (light orange) or without (dark orange) the administration of Venetoclax. Data points represent mean ± standard deviation from three biological replicates.

Table S1: Mass fraction of the different SwAb components measured by the SEC-MALS upon Venetoclax treatment. Switchable antibody/interleukin complexes were assembled *in vitro* with either the original LD3 or the variant 4 (LD3_v4), treated with venetoclax, and analyzed by size-exclusion chromatography coupled to multi-angle light scattering (SEC-MALS). The mass fraction of the different peaks shown in figures 1B, 2A, S1 and S3 were measured.

		Mass fraction (%)			
		Full complex	Partial complex	Bcl2-Fc	LD3-fused moiety
Ipilimumab	Original LD3	97%	N/D	3%	N/D
	LD3_v4	9.6%	N/D	36%	54.4%
αHER2	Original LD3	87.6%	N/D	1.4%	11%
	LD3_v4	7%	N/D	65.3%	27.7%
IL-15/IL-15R	Original LD3	62.1%	22.1%	5.2%	10.6%
	LD3_v4	N/D	N/D	62.8%	37.2%

Table S2: Amino acid sequences of the different proteins used. A stabilized version of Bcl2 fused to either human IgG1 (Bcl2-hmFc) or mouse IgG2 (Bcl2-mFc), a previously defined Bcl2-binding protein (LD3), an anti-CTLA4 Fab (Ipilimumab_H and Ipilimumab_L), a mouse interleukin 15 (mIL15), an anti-HER2 single chain fragment clone 4D5 (αHER2_scFv) and the same fused to an IgG (αHER2_Fc).

Bcl2-hmFc	AHAGRTGYDNREIVMKYIHYKLSQRGYEWDAGDDAEENRTEAPEGTESEVVHRALRDAGDDFERRYRRDF<br>AEMSSQLHLTPDTARQRFETVVEELFRDGVNWGRIVAFFEFGGVMCVESVNREMSPLVDNIAEWMTEYLNR<br>HLHTWIQDNGGWDAFVELYGPSMRGGGGSGTDKTHTCPPCPAPELLGGPSVFLFPPKPKDTLMISRTPEVT<br>CVVVDVSHEDPEVKFNWYVDGVEVHNAKTKPREEQYNSTYRVVSVLTVLHQDWLNGKEYKCKVSNKALPA<br>PIEKTISKAKGQPREPQVYTLPPSREEMTKNQVSLTCLVKGFYPSDIAVEWESNGQPENNYKTTPPVLDSDG<br>SFFLYSKLTVDKSRWQQGNVFSCSVMHEALHNHYTQKSLSLSPGKHHHHHH
Bcl2-mFc	AHAGRTGYDNREIVMKYIHYKLSQRGYEWDAGDDAEENRTEAPEGTESEVVHRALRDAGDDFERRYRRDF<br>AEMSSQLHLTPDTARQRFETVVEELFRDGVNWGRIVAFFEFGGVMCVESVNREMSPLVDNIAEWMTEYLNR<br>HLHTWIQDNGGWDAFVELYGPSMRGGGGSEPRVPITQNPCPPLKECPPCAAPDLLGGPSVFIFPPKIKDVLM<br>ISLSPMVTCVVVAVSEDDPDVQISWFVNNVEVHTAQTQTHREDYNSTLRVVSALPIQHQDWMSGKEFKCKV<br>NNRALPSPIEKTISKPRGPVRAPQVYVLPPPAEEMTKKEFSLTCMITGFLPAEIAVDWTSNGRTEQNYKNTAT<br>VLDSDGSYFMYSKLRVQKSTWERGSLFACSVVHEGLHNHLTTKTISRSLGKGTKHHHHHH
LD3	GQRWELALGRFLEYLSWVSTLSEQVQEELLSSQVTQELRALMDETMKELKAYKSELEEQLTPVAEETRARLS<br>KELQAAQARLGADMEDVRGRLVQYRGEVQAMLGQSTEELRVRLASHLIALQLRLIGDAFDLQKRLAVYQAG<br>A
Ipilimumab_H	QVQLVESGGGVVQPGRSLRLSCAASGFTFSSYTMHWVRQAPGKGLEWVTFISYDGNNKYYADSVKGRFTIS<br>RDNSKNTLYLQMNSLRAEDTAIYYCARTGWLGPFDYWGQGTLVTVSSASTKGPSVFPLAPSSKSTSGGTAA<br>LGCLVKDYFPEPVTVSWNSGALTSGVHTFPAVLQSSGLYSLSSVVTVPSSSLGTQTYICNVNHKPSNTKVDK<br>RVEPKSC
Ipilimumab_L	EIVLTQSPGTLSLSPGERATLSCRASQSVGSSYLAWYQQKPGQAPRLLIYGAFSRATGIPDRFSGSGSGTDF<br>TLTISRLEPEDFAVYYCQQYGSSPWTFGQGTKVEIKRTVAAPSVFIFPPSDEQLKSGTASVVCLLNNFYPREA<br>KVQWKVDNALQSGNSQESVTEQDSKDSTYSLSSTLTLSKADYEKHKVYACEVTHQGLSSPVTKSFNRGEC
mIL15	GTTCPPPVSIEHADIRVKNYSVNSRERYVCNSGFKRKAGTSTLIECVINKNTNVAHWTTPSLKCIRDPSLAGG<br>SGGSGGSGGSGGSGGSGGNWIDVRYDLEKIESLIQSIHIDTTLYTDSDFHPSCKVTAMNCFLLELQVILHEYS<br>NMTLNETVRNVLYLANSTLSSNKNVAESGCKECEELEEKTFTEFLQSFIRIVQMFINTSHHHHHH
αHER2_scFv	EVQLVESGGGLVQPGGSLRLSCAASGFNIKDTYIHWVRQAPGKGLEWVARIYPTNGYTRYADSVKGRFTISA<br>DTSKNTAYLQMNSLRAEDTAVYYCSRWGGDGFYAMDYWGQGTLVTVSSGGGGSGGGGSGGGGSDIQMT<br>QSPSSLSASVGDRVTITCRASQDVNTAVAWYQQKPGKAPKLLIYSASFLYSGVPSRFSGSRSGTDFTLTISSL<br>QPEDFATYYCQQHYTTPPTFGQGTKVEIK
αHER2_Fc	DYKDIVMTQSPSSLSASVGDRVTITCRASQDVNTAVAWYQQKPGKAPKLLIYSASFLYSGVPSRFSGSRSGT<br>DFTLTISSLQPEDFATYYCQQHYTTPPTFGQGTKVELKRATPSHNSHQVPSAGGPTANSGEVKLVESGGGLV<br>QPGGSLRLSCATSGFNIKDTYIHWVRQAPGKGLEWVARIYPTNGYTRYADSVKGRFTISADTSKNTAYLQMN<br>SLRAEDTAVYYCSRWGGDGFYAMDYWGQGTTVTVSSTGVHSEPRVPITQNPCPPLKECPPCAAPDLLGGP<br>SVFIFPPKIKDVLMISLSPMVTCVVVAVSEDDPDVQISWFVNNVEVHTAQTQTHREDYNSTLRVVSALPIQHQ<br>DWMSGKEFKCKVNNRALPSPIEKTISKPRGPVRAPQVYVLPPPAEEMTKKEFSLTCMITGFLPAEIAVDWTSN<br>GRTEQNYKNTATVLDSDGSYFMYSKLRVQKSTWERGSLFACSVVHEGLHNHLTTKTISRSLGKASGSRSLLA<br>NKRSEL
